# Comparison of physiological uptake of normal tissues in patients with cancer using ^18^F-FAPI-04 and ^18^F-FAPI-42 PET/CT

**DOI:** 10.3389/fnume.2022.927843

**Published:** 2022-09-29

**Authors:** Xingyu Mu, Xiaoxue Huang, Meng Li, Wenjie Sun, Wei Fu

**Affiliations:** Department of Nuclear Medicine, Affiliated Hospital, Guilin Medical University, Guilin, China

**Keywords:** ^18^F-FAPI, PET-CT, physiological uptake, normal tissues, cancer patients

## Abstract

**Purpose:**

To calculate the physiological uptake of various tissues in patients with cancer using ^18^F-AlF-NOTA-FAPI-04 (^18^F-FAPI-04) and ^18^F-AlF-NOTA-FAPI-42 (^18^F-FAPI-42) PET/CT and to compare the variation in standard uptake values between the two scans.

**Materials and methods:**

This retrospective analysis included 40 patients with cancer who underwent ^18^F-FAPI; the first 20 patients received ^18^F-FAPI-04 PET/CT and the remaining 20 patients received ^18^F-FAPI-42 PET/CT. A total of 49 normal tissues, including the brain (cerebrum/cerebellum), parotid and submandibular glands, palatine tonsils, and thyroid, were identified on CT images. For these normal tissues, maximum standardized uptake value (SUVmax) and mean standardized uptake value (SUVmean) were calculated. We also compared the SUVmean of identical tissues to explore the difference in biodistribution between the two radiotracers.

**Results:**

The accumulation of ^18^F-FAPI-04 and ^18^F-FAPI-42 showed an analogous pattern. High uptake of both radiotracers in the gallbladder, uterus, submandibular gland, and renal pelvis was demonstrated (range: SUVmax, 4.01–5.75; SUVmean, 2.92–4.22). Furthermore, the uptake of bony tissues was slightly higher in ^18^F-FAPI-42 than in ^18^F-FAPI-04 (range: SUVmean, 0.4 ± 0.22–0.9 ± 0.34 and 0.3 ± 0.24–0.7 ± 0.18, respectively, *p* < 0.05), while the uptake of some soft tissues was higher in ^18^F-FAPI-04 than in ^18^F-FAPI-42 (range: SUVmean, 0.9 ± 0.24–1.5 ± 0.35 and 0.9 ± 0.26–1.2 ± 0.37, respectively, *p* < 0.05).

**Conclusions:**

Both radioligands exhibited similar physiological uptake of normal tissues in patients with cancers. In addition, ^18^F-FAPI-42 demonstrated higher uptake of bone tissues than ^18^F-FAPI-04 while showing lower uptake of soft tissues than ^18^F-FAPI-04.

## Introduction

Carcinoma-associated fibroblasts (CAFs), a heterogeneous population of fibroblast-like cells, play a crucial role in tumor growth, migration, metastasis, extracellular matrix remodeling, therapy resistance, and immunosuppression (1). CAFs are genetically more stable and less susceptible to the development of resistance to therapy than cancer cells and are therefore excellent target cells for antitumor therapy (2). As a biological marker of CAFs, fibroblast activation protein (FAP), first described in 1986 as a cell surface antigen (3), is highly expressed in >90% of epithelial carcinomas, soft tissue sarcomas, granulation tissue, some fatal mesenchymal fibroblasts, and some benign conditions such as wound healing, fibrosis, arthritis, and atherosclerotic plaques (4, 5). For many years, efforts directed against FAP have been ineffective due to the absence of selective and potent inhibitors with properties suitable for clinical use. However, recent studies have provided promising strategies for drug development and potent FAP-selective inhibitors. In previous studies, ^68^Gallium- (^68^Ga-) DOTA-labeled-FAP inhibitor (FAPI) positron emission tomography/computed tomography (PET/CT) has been used as a diagnostic and therapeutic method in several tumors (6, 7).

Because ^68^Ga is usually eluted from a ^68^Ge–^68^Ga generator, a single synthesis will achieve only a small number of radiopharmaceuticals, enough for only two to four patients. Moreover, the short half-life of ^68^Ga (*T*_1/2_ = 68 min) limits the availability factor of radiotracers, especially in the transportation of radioactive drugs. ^18^Fluoride (^18^F), as the most widespread radionuclide, has already been applied in clinical practice and scientific research for several decades. It is beneficial due to its long half-life (*t*_1/2_ = 109.8 min), higher capacity, and lower positron energy. ^18^F can be considered an effective substitute for ^68^Ga to address the shortcomings of ^68^Ga-radiolabeled probes. Recently, a few studies have shown that ^18^F-FAPI has an effective detection capability in various types of cancers, such as lung cancer, signet-ring carcinoma, colon cancer, and hepatocellular cancer (HCC) (8, 9).

Although previous studies reported the biodistribution and standardized uptake value (SUV) of ^68^Ga-FAPI and ^18^F-FAPI (8, 10), they mainly focused only on a few major tissues, such as the brain, liver, kidney, or lungs. Cihan et al. determined the physical distribution of ^68^Ga-FAPI-04 in normal tissues and calculated the SUV for various organs (11). However, more extensive research is needed to determine the physical distribution of ^18^F-FAPI. Here, we calculate the SUV values of various normal tissues in patients with cancer using ^18^F-AlF-NOTA-FAPI-04 (^18^F-FAPI-04) PET/CT and ^18^F-AlF-NOTA-FAPI-42 (^18^F-FAPI-42) PET/CT and compared the SUVmean value between the two scans to provide a reference by which clinicians can better understand the biodistribution of the two radiotracers, thereby improving the diagnostic accuracy of ^18^F-FAPI PET/CT.

## Materials and methods

### Patients

This retrospective study was approved by the Institutional Review Board of the Affiliated Hospital of Guilin Medical University (Institutional Review Board Number: 2022WJWZCLL-01). All patients signed an informed consent form before participating, and all procedures were conducted in accordance with the tenets of the Helsinki Declaration. We randomly selected 40 patients with cancer who underwent ^18^F-FAPI PET/CT between September 2021 and March 2022: the first 20 patients received ^18^F-FAPI-04 PET/CT and the remaining 20 patients received ^18^F-FAPI-42 PET/CT.

### Pharmaceutical synthesis

Radiolabeling precursors with high chemical purity (>95%) were obtained from Jiangsu Huayi Technology Co., Ltd. (Jiangsu, China) and Beijing Paite Biotechnology Co., Ltd. (Beijing, China). ^18^F-FAPI-04 and ^18^F-FAPI-42 were produced in an automated synthesis module (GE and Allinone modules) as previously reported (12, 13). A detailed description of the radiosynthesis process and quality control protocol for ^18^F-FAPI-04/42 has been published elsewhere (12, 13). ^18^F-fluoride was produced *in situ* using the GE PET-trace 800 cyclotron system (GE, USA) by ^18^O-H2O irradiation with 16.5 MeV protons. Labeling efficiency and radiochemical purity were determined by radio thin layer chromatography (radio-TLC) (AR-2000, BIOSCAN, USA) and radio high-performance liquid chromatography (radio-HPLC) (UVIS-201, Alltech, USA). The radiochemical purity of the ^18^F-labeled FAPI-04/42 conjugates was ≥90%. Following HPLC-based quality control, 3.7 MBq (0.1 mCi)/kg of ^18^F-FAPI-04/42 was injected.

### PET/CT protocol

All images were obtained with the Ingenuity TF PET/CT (Philips, Amsterdam, The Netherlands). After injection, whole body images were taken from the vertex to mid-thigh during the 1st hour. After CT imaging (CT parameters: 120 kV, 250 mAs/slice, 600-mm trans axial FOV, no gap, 64 × 0.625 mm collimation, pitch 0.8, 0.75 s rotation time, 1 mm slice thickness, and 512 × 512 matrix), PET images (PET parameters: 3D FOV 20 cm, ordered subset expectation-maximization algorithm [OSEM] 3 iterations/12 subset, and full width at half maximum [FWHM] at 3 mm) were taken bedside at 2.5 min in the same position to include the same regions. All patients were asked to drink water and to urinate immediately before PET/CT imaging.

### Image analysis

All ^18^F-FAPI PET/CT images were reviewed on the MedEx (MedEX Technology Ltd. Co., Beijing, China) workstation for registration, fusion, and measurement. Two experienced nuclear medicine physicians identified the organs on the CT images: the brain (cerebrum/cerebellum), parotid and submandibular glands, palatine tonsils, thyroid, lymph nodes (if present), breasts, lungs, thymus, left ventricular walls, mediastinal blood pool, vertebral bone marrow, liver, spleen, pancreas (tail/corpus), stomach, small and large intestine, adrenal glands, kidneys, uterus, testes, prostate, muscles, and bones. SUVmax values of the primary tumor, if any, were also measured. SUVmax of healthy tissues was calculated from tumor-free areas originating from areas not suggestive of primary tumor or metastasis on ^18^F-FAPI PET/CT. Areas of interest were drawn from tissues on the right side of the body, except for metastases in symmetrical tissues. The volumes of interest (VOIs) were drawn in three consecutive slices on the PET images centered on the maximum voxel value for the mentioned organs, and the maximum and mean values of the SUV in the VOIs were recorded. VOIs were always placed within the limits of the activity distribution to minimize a partial volume effect. VOIs were delineated at 1 cm for small tissues and at 2 cm for large organs such as the liver and lungs.

### Statistical analysis

SPSS version 25.0 (IBM SPSS Corporation; Armonk, NY, USA) was used for statistical analyses. The median SUVmax and SUVavg values of the organs were calculated. The differences in SUVmax and SUVmean between ^18^F-FAPI-42 and ^18^F-F-FAPI-04 were evaluated using the independent sample *t*-test (variables with normal distribution) or the Mann–Whitney *U* test (variables without normal distribution). Two-tailed *p*-values < 0.05 were considered indicative of a statistically significant difference.

## Results

Patients' clinical and demographic characteristics are presented in Table 1. All patients tolerated these examinations well. There were no drug-related pharmacological effects or physiologic responses. During the injection and until the end of examination, no patient complained of any symptoms or developed any adverse effects.

**Table 1:** Patient characteristics.

**Population characteristics Patients (*n*) = 40, sex = M (15), F (25)**	**Scan 1 (FAPI-04)**			**Scan 1 (FAPI-42)**		
	**Mean ±SD**	**Median**	**Range**	**Mean ±SD**	**Median**	**Range**
Age	57 ± 12	57	32–82	54 ± 11	56	32–76
Weight	58 ± 11	56	44–82.5	57 ± 7	56	46–75
Administerted activity(Mbq)	251 ± 49	255.3	188.7–355.2	228 ± 54	223.85	177.6–370
Uptake time(min)	71 ± 11	68.5	54–90	69 ± 13	69	52–91

### Uptake of normal organ tissues using ^18^F-FAPI-04 and ^18^F-FAPI-42 PET/CT

**Figure 1:**
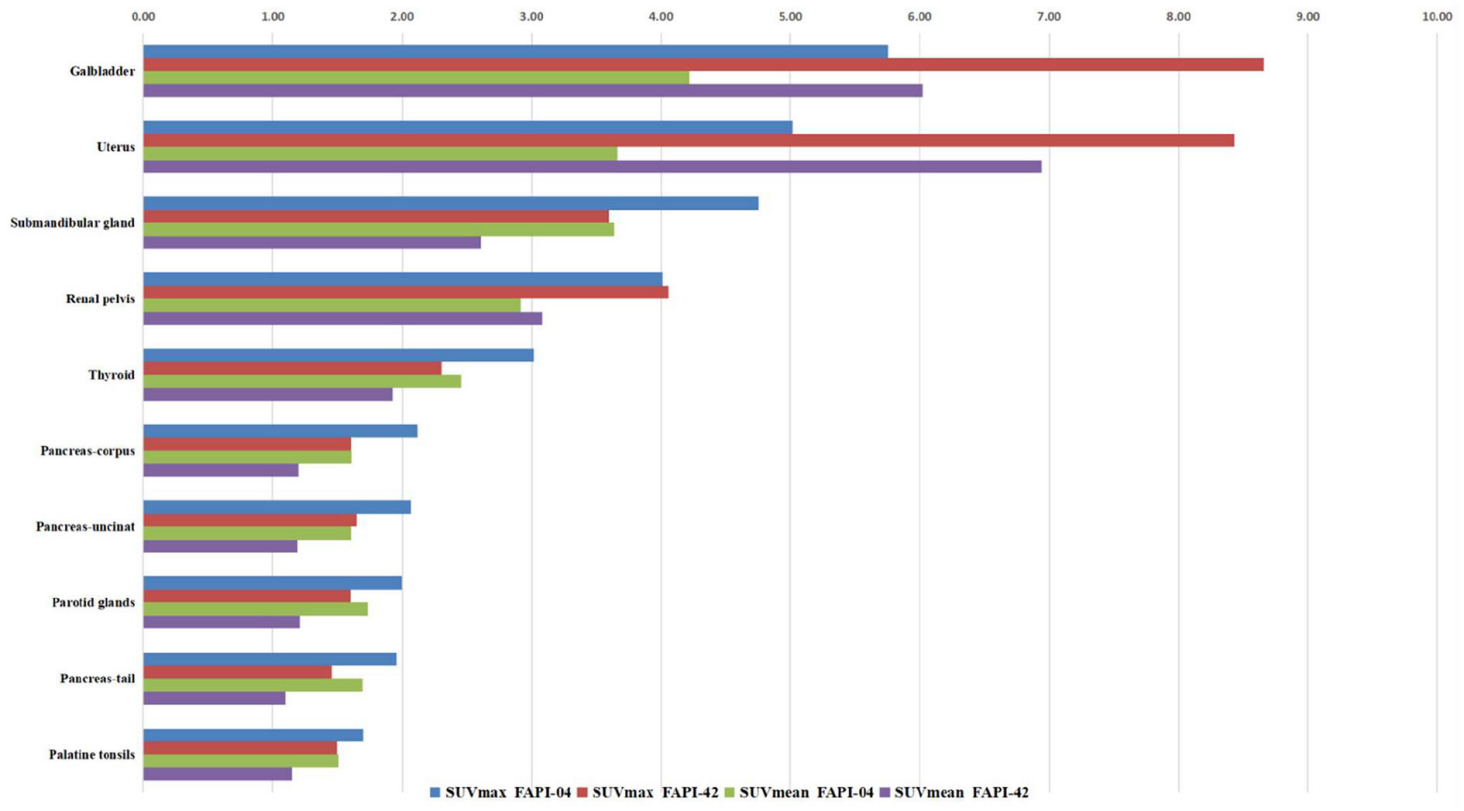
Median maximum standardized uptake value (SUVmax) and SUVmean in most tissues and organs.

The distribution of two types of radiotracer in various organs is shown in Figure 1, Supplementary Table S1, and Supplementary Figure S1. In the ^18^F-FAPI-04 cohort, we found that the gallbladder, uterus, submandibular gland, and renal pelvis exhibited high uptake in the median of SUVmax and SUVmean (range: 4.01–5.75 and 2.92–4.22, respectively). In addition, the rest of the organs showed moderate-to-low uptake in the median of SUVmax and SUVmean. However, the cerebral cortex and cerebellum showed almost no ^18^F-FAPI-04 uptake in SUVmax and SUVmean (range: 0.19–0.23 and 0.14–0.14, respectively). In contrast, the gallbladder, uterus, submandibular gland, and renal pelvis showed high ^18^F-FAPI-42 uptake in the median of SUVmax and SUVmean (range: 3.6–8.8 and 2.6–6.4, respectively). Although the cerebral cortex and cerebellum did not have similar ^18^F-FAPI-42 uptake in SUVmax and SUVmean (range: 0.19–0.21 and 0.12–0.14, respectively), ^18^F-FAPI-42 uptake in other organs is slightly different from ^18^F-FAPI-04 uptake. For example, the median of SUVmean for the testis, pancreas head, supraclavicular fatty tissues, and other tissues was much higher in ^18^F-FAPI-42 than in ^18^F-FAPI-04.

### Variation of SUV in healthy tissues

**Figure 2:**
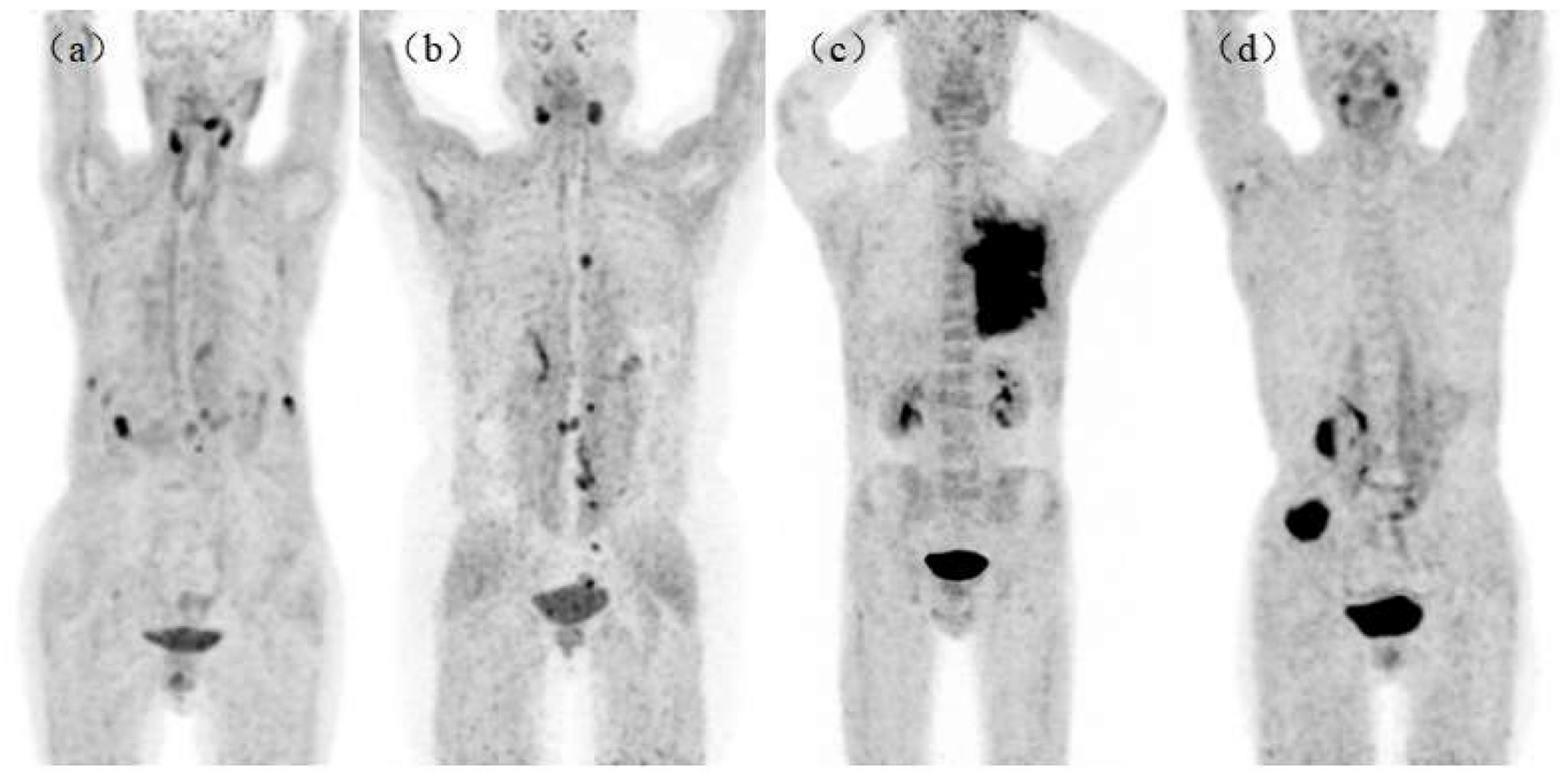
Maximum intensity projections (MIPs) in patients with lung cancer [**(a)**, ^18^F-AlF-NOTA-FAPI-04 (^18^F-FAPI-04)], ovarian cancer [**(b)**, ^18^F-FAPI-04], lung cancer [**(c)**, ^18^F-AlF-NOTA-FAPI-42 (^18^F-FAPI-42)], and colon cancer [**(d)**, ^18^F-FAPI-42]. All patients had no bony metastasis and received bone treatment before receiving scans.

**Figure 3:**
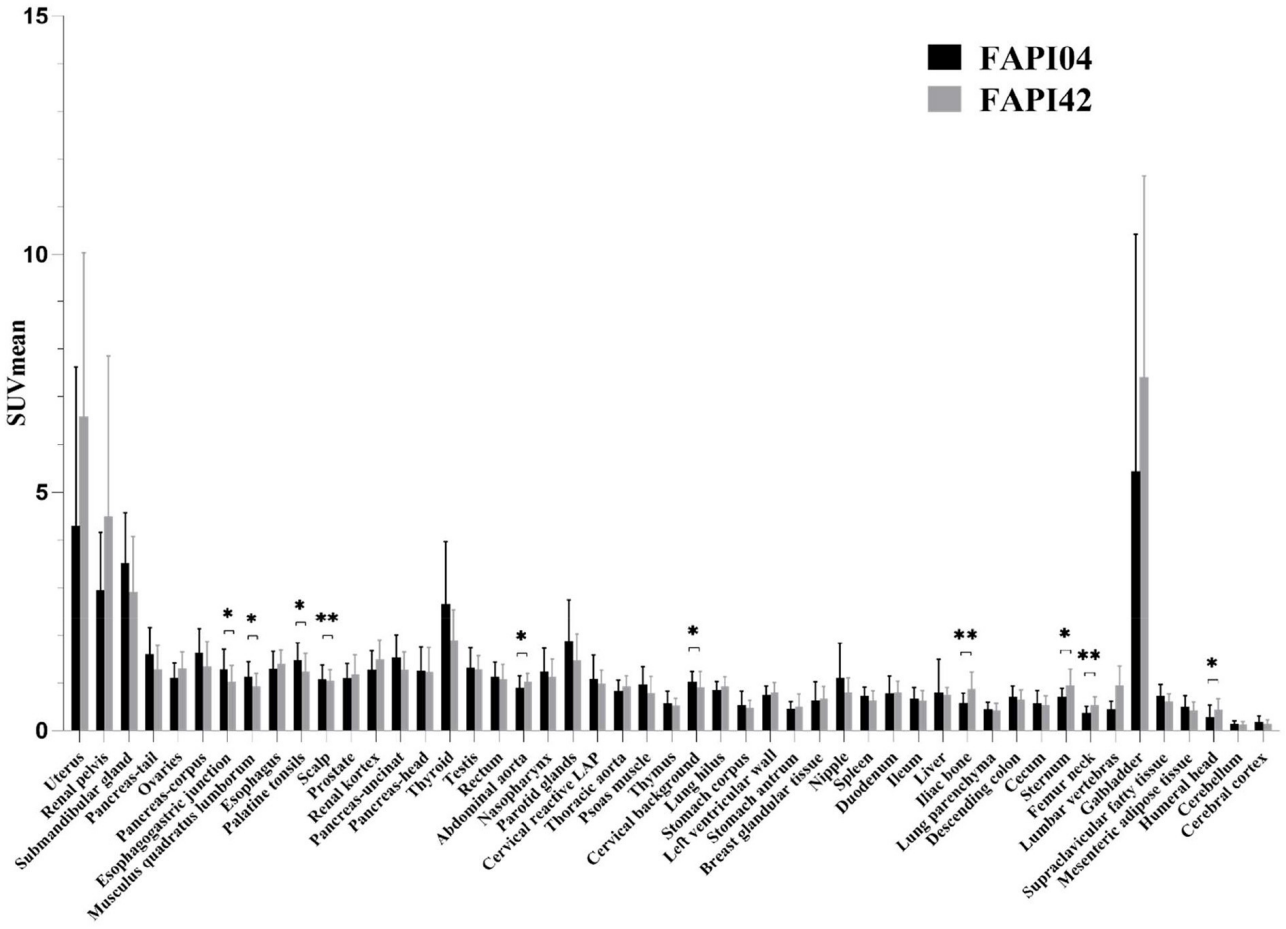
Comparison of SUVmean between ^18^F-FAPI-04 and ^18^F-FAPI-42 positron emission tomography/computed tomography (PET/CT) imaging. *p < 0.05, **p < 0.01.

We found no significant changes in age, weight, administered injection activity, and uptake time after tracer administration between the two scans (*p* = 0.759, 0.848, 0.171, and 0.551, respectively). Significant differences were observed in the scalp, cervical background, palatine tonsils, esophagogastric junction, musculus quadratus lumborum, abdominal aorta, sternum, humeral head, iliac bone, and femoral neck for the SUVmean value between ^18^F-FAPI-04 and ^18^F-FAPI-42 (range: 0.3 ± 0.24–1.3 ± 0.41 and 0.4 ± 0.22–1.2 ± 0.37, *p* < 0.05) (Table 2, Supplementary Table S1). However, there was no significant bias (*p* > 0.05) in other tissues such as pancreas-tail, pancreas-uncinate, and renal pelvis (Table 2). Similarly, bony SUVmax in ^18^F-FAPI-42 was higher than that in ^18^F-FAPI-04 (range: 0.62 ± 0.32–1.26 ± 0.48; 0.39 ± 0.31–0.91 ± 0.23, *p* < 0.05). Moreover, the SUVmax of the pancreas-uncinate and psoas muscle in ^18^F-FAPI-04 presented statistically higher uptake than that in ^18^F-FAPI-42 (2.02 ± 0.56 and 1.23 ± 0.46; 1.66 ± 0.45 and 1.05 ± 0.63; and *p* = 0.028 and 0.037, respectively). The representative imaging of cancer patients were presented in Figure 2. All distribution differences between two radiotracers were shown in Figure 3.

**Table 2:** Comparison between mean standardized uptake value (SUVmean) values observed in this study and values obtained by Wang et al.

**Healthy tissue**	**Our result**		**Wang et al.'s result**
	**^18^F-FAPI-04**	**^18^F-FAPI-42**	**^18^F-FDG**
Galbladder	5.4 ± 2.86	7.4 ± 4.1	1.24 ± 0.45
Submandibular gland	3.5 ± 1.02	2.9 ± 1.14	2.22 ± 0.77
Thyroid	2.7 ± 1.26	1.9 ± 0.62	1.45 ± 0.57
Pancreas-corpus	1.6 ± 0.49	1.4 ± 0.50	1.48 ± 0.33
Parotid glands	1.9 ± 0.83	1.5 ± 0.54	1.75 ± 0.79
Palatine tonsils	1.5 ± 0.35	1.2 ± 0.37	4.08 ± 1.51
Testis	1.3 ± 0.38	1.2 ± 0.28	2.73 ± 0.60
Nasopharynx	1.2 ± 0.48	1.1 ± 0.36	1.66 ± 0.83
Esophagus	1.3 ± 0.36	1.4 ± 0.27	1.61 ± 0.61
Rectum	1.1 ± 0.30	1.1 ± 0.32	1.58 ± 0.79
Prostate	1.1 ± 0.29	1.2 ± 0.39	1.9 ± 0.37
Liver	0.8 ± 0.68	0.7 ± 0.13	2.06 ± 0.45
Lung hilus	0.9 ± 0.16	0.9 ± 0.18	1.33 ± 0.32
Spleen	0.7 ± 0.19	0.6 ± 0.20	1.77 ± 0.38
Breast glandular tissue	0.6 ± 0.37	0.7 ± 0.25	0.57 ± 0.32
Stomach corpus	0.5 ± 0.28	0.5 ± 0.14	1.87 ± 0.82
Stomach antrum	0.5 ± 0.13	0.5 ± 0.25	1.57 ± 0.6
Cecum	0.6 ± 0.27	0.5 ± 0.19	1.08 ± 0.35
Lumbar vertebras	0.4 ± 0.16	0.9 ± 0.40	1.61 ± 0.47
Cerebellum	0.1 ± 0.06	0.1 ± 0.05	8.22 ± 2.40

## Discussion

The objectives of this study were to access the distribution pattern of healthy tissues using ^18^F-FAPI-04 and ^18^F-FAPI-42 PET/CT and the variation of healthy tissues by SUV parameters in two types of radioligands in patients with cancer. We found a statistically significant bias in the scalp, cervical background, palatine tonsils, esophagogastric junction, musculus quadratus lumborum, abdominal aorta, sternum, humeral head, iliac bone, and femoral neck for the SUVmean parameters, indicating significant differences in these tissues.

Our results demonstrated high uptake of ^18^F-FAPI-04 and ^18^F-FAPI-42 in the gallbladder, uterus, submandibular gland, renal pelvis, and thyroid. Among these tissues, the gallbladder and renal pelvis served as the pathway of excretion for the two radiotracers. A possible explanation for this could be the different lipophilicities of the NOTA- and DOTA-chelator groups, which caused extra excretion in the biliary system (14). Compared with ^68^Ga-FAPI PET/CT, the shortcoming of ^18^F-FAPI likely influences the detection of lesions in these regions, especially in the case of thyroid diseases and tumors of the pancreas, gallbladder, and biliary tract (15, 16). Moderate FAP expression has been shown in other cervical, respiratory, abdominal, and pelvic organs, which is in agreement with studies previously conducted with ^68^Ga-FAPI and ^18^F-FAPI PET/CT (7, 11, 17). Low uptake of ^18^F-FAPI-04 and ^18^F-FAPI-42 has been demonstrated in the cerebral cortex and cerebellum because the brain has low FAP expression and the radiotracer cannot cross the blood–brain barrier (18). However, in both the scans, higher uptake was demonstrated in the scalp than in other regions/organs. Thus, we can infer that both the radiotracers have the advantage in assessing brain malignancies because they can provide an excellent tumor-background ratio in the detection of these regional tumors.

The results of the comparison of SUVmean for various tissues between ^18^F-FAPI-04 and ^18^F-FAPI-42 revealed that some organs exhibited differences in biodistribution. First, bone, including iliac bone, femoral neck, sternum, humeral head, and lumbar vertebrae, demonstrated higher uptake of ^18^F-FAPI-42 than ^18^F-FAPI-04, which is in accordance with preclinical mice models (14), despite the fact that lumbar vertebrae did not show a significant bias of SUVmean between the two radiotracers. Hence, it can be speculated that ^18^F-FAPI-42 is weaker than ^18^F-FAPI-04 in detecting bone diseases, as a result of poor tumor-background ratio caused by ^18^F-FAPI-42. Additionally, physicians in our department complained that higher uptake of soft tissues was demonstrated in ^18^F-FAPI-04 than in ^18^F-FAPI-42. Indeed, high uptake of some soft tissues, such as the cervical background, musculus quadratus lumborum, and esophagogastric junction, is demonstrated in ^18^F-FAPI-04, which signifies that ^18^F-FAPI-42 is superior for identifying lesions in these regions. High uptake of the palatine tonsils in ^18^F-fluoro-2-deoxy-D-glucose (^18^F-FDG) was frequently observed due to the physical uptake or inflammation in the tonsils, which confused a variety of nuclear physicians to diagnose diseases in this area during clinical practice. Notably, compared with ^18^F-FDG PET/CT, both ^18^F-FAPI-04 and ^18^F-FAPI-42 have lower uptake in the palatine tonsils (19). Furthermore, our results indicated that, compared with ^18^F-FAPI-42, ^18^F-FAPI-04 can support lower uptake in the palatine tonsils, which means that ^18^F-FAPI-04 can ensure excellent tumor-background ratio in detecting lesions of the palatine tonsils. This result may assist physicians make correct decisions about the diagnosis of diseases in the palatine tonsils. It has been suggested that in the parotid and salivary glands, thyroid, and pancreas, the accumulation of ^68^Ga-FAPI-04 is less than that ^18^F-FAPI-42 (8). This does not appear to be the case in our study, and this discrepancy can be attributed to the chelator, the isotope, and the organs included. Intense uptake of radioactivity was also observed in the gallbladder and common bile duct, which was similar in the two studies. In addition, moderate and mild uptake of radioactivity was similarly observed in other organs. These findings are unexpected and thus suggest that FAP binding capacity is diverse among these radiotracers or radioligands. Further research is needed to investigate this discrepancy.

^18^F-fluoro-2-deoxy-D-glucose PET/CT is a brilliant tool for diagnostic oncologic imaging, as malignant lesions have high glucose utilization, before FAPI-PET was reported. As the University Hospital Heidelberg group developed a series of quinoline-based FAPIs based on clinical and preclinical research, it showed promising performance in oncology management in small-sample studies (6, 7, 20). It was endowed with great expectation of being an auspicious tool for the replacement or supplementation of ^18^F-FDG in the diagnosis and staging of tumors, as well as in the evaluation of efficacy and prognosis in various types of cancers. However, a difference in the uptake of ^18^F-FDG and ^18^F-FAPI in a variety of organs was insufficient. Thus, when reviewing the results of our study with the result of a previous study (21) (characterizing the physical uptake of ^18^F-FDG in normal tissues), we found that there are abundant differences in biodistribution between ^18^F-FAPI and ^18^F-FDG (Table 2). In most normal tissues (14 of 20 organs), ^18^F-FAPI uptake was lower than ^18^F-FDG uptake. Therefore, identifying which radiotracer can provide a low level of uptake in identical organs is indispensable, it is recommended to improve image interpretation skills, familiarity with the normal pattern, and intensities of the different types of radiotracers. For example, compared with ^18^F-FDG, ^18^F-FAPI has a low level of uptake in the brain. Hence, ^18^F-FAPI has superiority in detecting malignant tumor metastases in the brain, which has been proven in lung cancer by Wang et al. (22). In contrast, the SUVmean of the pancreas, muscles, and submandibular and parotid glands was slightly higher on ^18^F-FAPI-04 PET/CT than on ^18^F-FDG PET/CT. However, a few studies have described FAPI-PET as a robust method to diagnose pancreatic and head-and-neck cancers (10, 23), which means that a slight difference of SUVmean between ^18^F-FAPI and ^18^F-FDG cannot affect the accuracy of diagnosis in these types of cancer.

Giesel et al. investigated and compared organ biodistribution and tumor uptake between ^68^Ga-FAPI and ^18^F-FDG PET/CT in patients with various types of cancers, but did not analyze the advantages of the FAPI-PET ligand in detecting different types of tumors (24). Cihan et al. (11) only determined the physiological uptake and SUVmax of ^68^Ga-FAPI-04 in various tissues and organs. Similarly, they did not compare the variation of SUV in different FAPI-PET ligands. In our study, we found an approximate biodistribution in two ligands, although minor differences were exhibited in two ligands, such as bone, cervical background, and muscle. Overall, our study provided a guide to understanding a variation in the uptake of ^18^F-FAPI-04 and ^18^F-FAPI-42 for normal tissues. This is an important aspect to assist physicians select more effective radiotracers for different types of cancers, to improve sensitivity, specificity, and accuracy in diagnosis, staging, and restaging in patients with cancer.

This study has some limitations. First, the sample size and the number of patients in each cancer group were small. This could limit the scope of our conclusion. Second, we did not analyze the uptake of tumor lesions and the tumor-to-background ratio between the two radiotracers because the population of both groups was diverse. Third, the whole organ was not included in the VOI, which only included 1 or 2 cm per organ. Finally, we only enroll patients with cancer because these radiopharmaceuticals are relatively new and cannot be applied to a healthy person. Despite these limitations, we propose that this study certainly adds to our understanding of the biodistribution of these two tracers. Thus, further studies are needed to address these limitations.

## Conclusion

In this retrospective study, to better understand the biodistribution features of both tracers, we investigated the physiological uptake patterns of FAPI in normal tissues throughout the body using ^18^F-FAPI-04 and ^18^F-FAPI-42 PET/CT images. We observed discrepancies between the two radiotracers in physiological uptake patterns; this has the potential to improve interpretive accuracy if used to complement the clinician's expertise in reviewing ^18^F-FAPI-PET/CT images and choosing the appropriate ligands to image different types of cancers.

## Data availability statement

The original contributions presented in the study are included in the article/Supplementary material, further inquiries can be directed to the corresponding author/s.

## Ethics statement

The studies involving human participants were reviewed and approved by Institution Review Board of the Affiliated Hospital of Guilin Medical University. The patients/participants provided their written informed consent to participate in this study. Written informed consent was obtained from the individual(s) for the publication of any potentially identifiable images or data included in this article.

## Author contributions

WF and XM contributed to the conception and design of the study. XH collected and analyzed the data. WS and XM performed statistical analyses. XM wrote the first draft of this manuscript. ML, XH, and WS wrote sections of the manuscript. WS revised the manuscript. All authors contributed to manuscript revision and read and approved the submitted version.

## Funding

This work was financially supported by Guangxi Health Commission, No. Z20200762.

## Conflict of interest

The authors declare that the research was conducted in the absence of any commercial or financial relationships that could be construed as a potential conflict of interest.

## Publisher's note

All claims expressed in this article are solely those of the authors and do not necessarily represent those of their affiliated organizations, or those of the publisher, the editors and the reviewers. Any product that may be evaluated in this article, or claim that may be made by its manufacturer, is not guaranteed or endorsed by the publisher.
