# Supplementary figures and images for: Comparison of physiological uptake of normal tissues in patients with cancer using 18F-FAPI-04 and 18F-FAPI-42 PET/CT
Source: Front Nucl Med. 2022 Sep 29;2:927843. doi: 10.3389/fnume.2022.927843 (PMC11440963; doi:10.3389/fnume.2022.927843)

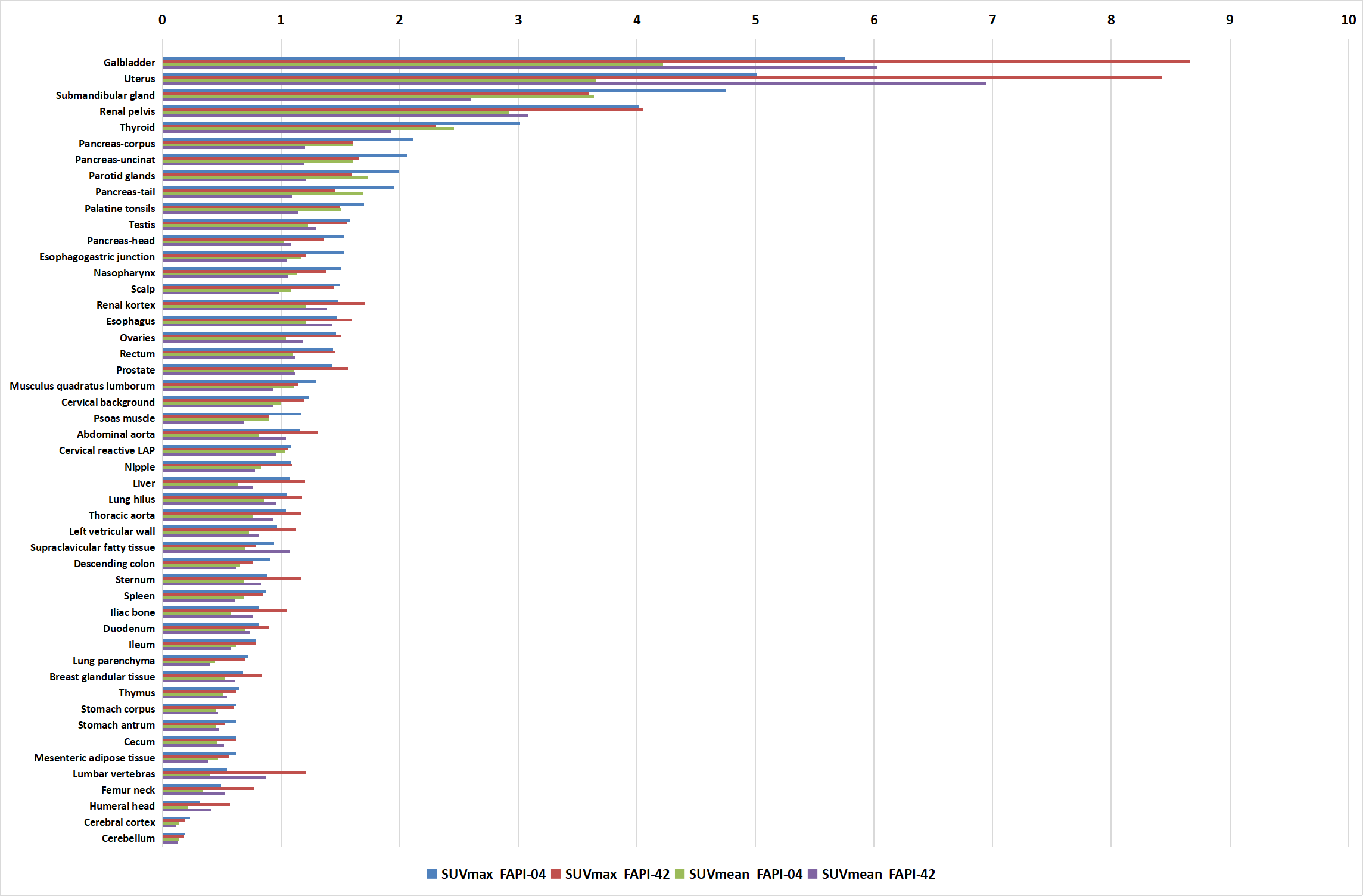

Supplement: Supplementary file 2 [file Image_1.tif]
